# Investigation of mesalazine as an antifibrotic drug following myocardial infarction in male mice

**DOI:** 10.14814/phy2.15809

**Published:** 2023-09-09

**Authors:** Stephan R. Künzel, Luise Winter, Maximilian Hoffmann, Theresa A. Kant, Jessica Thiel, Romy Kronstein‐Wiedemann, Erik Klapproth, Kristina Lorenz, Ali El‐Armouche, Susanne Kämmerer

**Affiliations:** ^1^ Institute of Pharmacology and Toxicology, Faculty of Medicine Carl Gustav Carus, Technische Universität Dresden Dresden Germany; ^2^ Institute of Transfusion Medicine, Faculty of Medicine Carl Gustav Carus, Technische Universität Dresden Dresden Germany; ^3^ German Red Cross Blood Donation Service North‐East Dresden Germany; ^4^ Institute of Pharmacology and Toxicology, Julius‐Maximilians‐University of Würzburg Würzburg Germany; ^5^ Leibniz‐Institut für Analytische Wissenschaften ‐ISAS‐ e.V Dortmund Germany

**Keywords:** drug repurposing, fibrosis, mesalazine, myocardial infarction

## Abstract

**Objectives:**

Myocardial infarction (MI) initiates a complex reparative response during which damaged cardiac muscle is replaced by connective tissue. While the initial repair is essential for survival, excessive fibrosis post‐MI is a primary contributor to progressive cardiac dysfunction, and ultimately heart failure. Currently, there are no approved drugs for the prevention or the reversal of cardiac fibrosis. Therefore, we tested the therapeutic potential of repurposed mesalazine as a post‐MI therapy, as distinct antifibrotic effects have recently been demonstrated.

**Methods:**

At 8 weeks of age, MI was induced in male C57BL/6J mice by LAD ligation. Mesalazine was administered orally at a dose of 100 μg/g body weight in drinking water. Fluid intake, weight development, and cardiac function were monitored for 28 days post intervention. Fibrosis parameters were assessed histologically and via qPCR.

**Results:**

Compared to controls, mesalazine treatment offered no survival benefit. However, no adverse effects on heart and kidney function and weight development were observed, either. While total cardiac fibrosis remained largely unaffected by mesalazine treatment, we found a distinct reduction of perivascular fibrosis alongside reduced cardiac *collagen* expression.

**Conclusions:**

Our findings warrant further studies on mesalazine as a potential add‐on therapy post‐MI, as perivascular fibrosis development was successfully prevented.

## 1 INTRODUCTION

Myocardial infarction (MI) is among the leading causes of mortality worldwide (Roth et al., 2020). Despite tremendous advances in the acute treatment, chronic heart failure remains a regular long‐term complication (Talman & Ruskoaho, 2016). Ischemic injury results in the irreversible loss of cardiomyocytes, which only have limited capacity for endogenous renewal (Gibb et al., 2020; Talman & Ruskoaho, 2016). In order to prevent ventricular rupture, activated cardiac fibroblasts replace damaged myocardial tissue with a fibrotic scar (Gibb et al., 2020; Humeres & Frangogiannis, 2019; Talman & Ruskoaho, 2016). During this process, fibroblasts undergo a phenotype conversion towards their activated, highly secretory myofibroblast phenotype (Poulet et al., 2016). Although this reparative response is crucial upon acute injury, uncontrolled pan‐ventricular fibroblast activation (Nagaraju et al., 2017) due to persistent TGFβ secretion, increased mechanic stress and inflammation (Humeres & Frangogiannis, 2019) lead to progressive interstitial and perivascular fibrotic remodeling and ultimately heart failure (Fu et al., 2018; Rog‐Zielinska et al., 2016; Talman & Ruskoaho, 2016). Consequently, cardiac fibrosis is an attractive pharmacological target for heart failure therapies. However, as no currently available drugs can prevent or reverse cardiac fibrotic remodeling (Hoffmann et al., 2021; Zhao et al., 2020), effective antifibrotic drug therapy remains an unmet challenge. Compared to de novo drug development, repurposing, that is, identification of new therapeutic uses for existing drugs, could offer several benefits, including reduced development time, lower costs, and potentially improved safety profiles (Pushpakom et al., 2019). Mesalazine (5‐aminosalicylic acid) is a drug primarily used to treat inflammatory bowel disease (Bantel et al., 2000; Brogden & Sorkin, 1989; Hoffmann et al., 2021), inhibiting the production of pro‐inflammatory cytokines and chemokines by immune cells and fibroblasts, as well as the activation of nuclear factor‐kappa B (NFκB), which plays a key role in the inflammatory response (Bantel et al., 2000; Hoffmann et al., 2021). Previous research has identified distinct antifibrotic effects of mesalazine in the heart, the liver and the skin (Hoffmann et al., 2021; Künzel et al., 2021; Newe et al., 2021; Ramadan et al., 2018). Thus, mesalazine appears to be an ideal candidate for antifibrotic drug repurposing after MI, as it can potentially target both the inflammatory and fibrotic pathways that contribute to fibrosis‐driven cardiac dysfunction (Francis Stuart et al., 2016; Gibb et al., 2020). Furthermore, mesalazine has a well‐established safety profile and has been approved for clinical use for decades (Beiranvand, 2021; Ye & van Langenberg, 2015), which could expedite its translation to clinical trials for the treatment of fibrosis. In the present study, we investigated the effects of systemic mesalazine treatment on cardiac fibrosis and heart function following MI.

## 2 METHODS

The sample size was not predetermined statistically due to the exploratory nature of this study. The animals were randomly allocated to the experimental groups. The investigators were blinded during outcome assessment.

### 2.1 Animals

Animal experiments were authorized by *Landesdirektion Sachsen*, Dresden, Germany, according to the German animal welfare regulations (TVV 64/2018) and complied with the ARRIVE guidelines as well as the guidelines from Directive 2010/63/EU on the protection of animals used for scientific purposes. For this study, male wild‐type C57BL/6J mice were obtained from Janvier Laboratories, Saint Berthevin Cedex, France.

### 2.2 Myocardial infarction and subsequent mesalazine treatment

**FIGURE 1:**
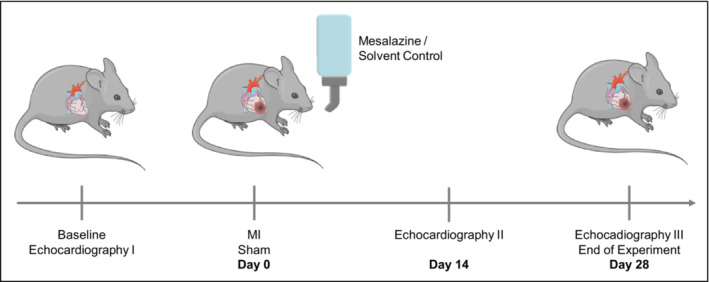
Experimental protocol of the study. Male C57BL/6J mice were randomly allocated to the experimental groups: Sham (control, *N* = 8), MI (*N* = 11), MI and subsequent mesalazine treatment (*N* = 11; 100 μg mesalazine per g body weight). Echocardiography was performed at baseline, 14 and 28 days after MI/ Sham. At the end of the experiment, all remaining mice were sacrificed for subsequent histological and gene expression analysis. Images for this figure were modified from Servier Medical Art, licensed under a Creative Commons Attribution 3.0 Unported License. http://smart.servier.com/.

At the age of 8 weeks, left anterior descending coronary artery ligation or Sham operation were performed as described previously (Klapproth et al., 2022). The following week, mice were monitored twice daily and subsequently three times a week. Mesalazine (100 μg/g body weight; Fisher Scientific GmbH, Schwerte, Germany, catalogue number: 11466846) was administered via the drinking water. Treatment started directly after MI induction. Control animals received solvent control (the volume of 20% hydrochloric acid equal to the mesalazine solution were added to the water). The pH of the drinking water was monitored and was between 5 and 6. Mice were weighed at baseline and once a week after MI to adjust drug dosage based on an estimated daily fluid intake of 4 mL. Since mesalazine precipitates in light, the water bottles were wrapped in tin foil. Echocardiography was performed at baseline, 14 days after MI and at the end of the experiment after 28 days. After the final echocardiography, animals were sacrificed by cervical dislocation. Subsequently, blood and tissue samples were collected. A schematic overview of the experimental procedure is given in Figure 1.

### 2.3 Echocardiography

Echocardiography was performed using a Vevo 3100 system (FUJIFILM VisualSonics) as described previously (Klapproth et al., 2022; Künzel et al., 2021). Briefly, animals were anesthetized with 1.5% v/v isoflurane. Surface ECG and body temperature were obtained continuously. The chest area was shaved using depilatory cream. A MX400 transducer was positioned to obtain 2D B‐mode parasternal long and short axis views and M‐mode view. Echocardiographic analysis was performed using the Vevo 2.1.0 software (FUJIFILM VisualSonics).

### 2.4 Histology and image analysis

Mid‐ventricular heart sections were immediately embedded in OCT Embedding Matrix (cat. no. 6478.1, Carl Roth GmbH + Co. KG, Karlsruhe, Germany) at −10°C. Sectioning and subsequent Sirius red staining were performed by the histology facility at the Center for Molecular and Cellular Bioengineering (CMCB), Dresden. Images were acquired with a Keyence BZ‐X710 microscope (Keyence Corporation of America). Quantification of fibrotic areas in relation to the total tissue area was done using the FIJI 1.52n software (Newe et al., 2021; Schindelin et al., 2012).

### 2.5 RNA isolation, cDNA synthesis and qPCR

SYBR green (cat. no. 1725270, Bio‐Rad Laboratories GmbH) real‐time PCR was performed in a CFX96 Touch Deep Well Real‐Time PCR detection system (Bio‐Rad Laboratories GmbH) to measure target gene expression. Ready‐to‐use primers were purchased from Bio‐Rad (Bio‐Rad Laboratories GmbH, Munich, Germany, Table 1). Hypoxanthine phosphoribosyltransferase 1 (HPRT) was used as a housekeeping gene. Catalogue numbers of the primers used in this study are provided in Table 1. mRNA was isolated with a RNeasy Micro Kit (cat. no. 74004, Qiagen). cDNA was synthesized using the iScript Advanced cDNA synthesis kit (cat. no. 1725037, Bio‐Rad Laboratories GmbH). CFX manager software (Bio‐Rad Laboratories GmbH) was used for data analysis.

**TABLE 1:** Primer catalogue numbers.

Primer	Catalogue number
COL1A1, Mouse	qMmuCEP0052648
COL1A2, Mouse	qMmuCIP0033742
COL3A1, Mouse	qMmuCIP0029022
HPRT, Mouse	qMmuCEP0054164

### 2.6 Statistical analysis

All graphic results are presented as mean ± SD. Graph Pad Prism v.9 (GraphPad Software) was used for statistical analysis and figure preparation. All datasets were tested for normality. For comparisons of 3 groups, 1‐way ANOVA or Kruskal‐Wallis test were performed with Holm‐Šídák or Dunn posttest, respectively. For survival analysis a Log‐rank (Mantel‐Cox) test was performed. *p* < 0.05 was considered statistically significant.

## 3 RESULTS

### 3.1 Mesalazine effects on survival and cardiac function after MI

**FIGURE 2:**
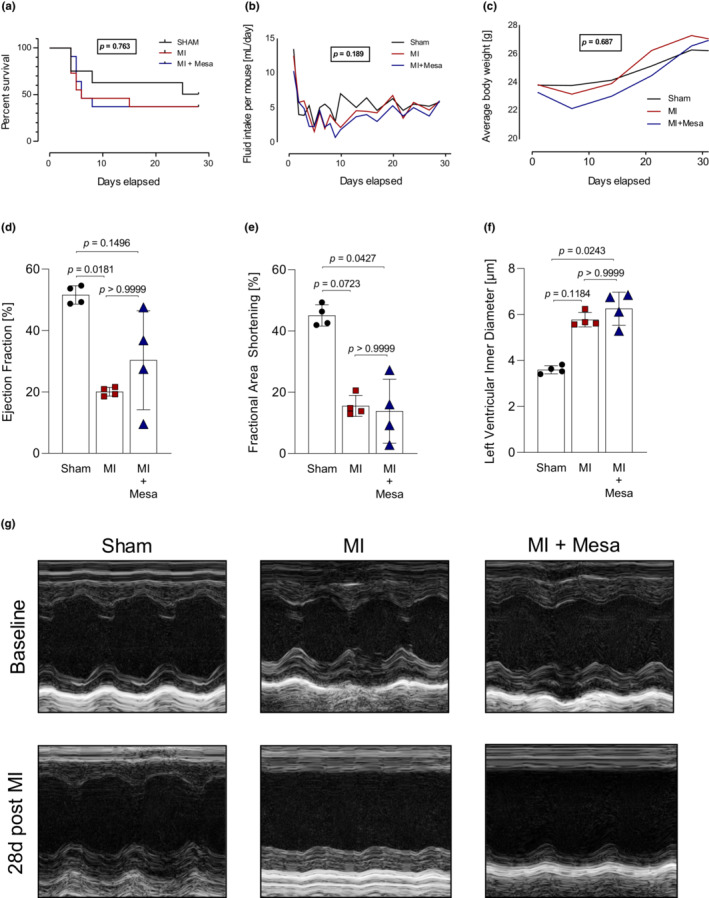
Mesalazine effects on survival, weight development and cardiac function following MI. The bar graphs represent the mean values of the indicated study populations normalized to the mean value of the respective control group ± SD. (a) Survival curves after MI over a period of 28 d. Animals at the beginning of the experiment: Sham = 8, MI = 11, MI + Mesalazine = 11. *p*‐values were determined by Log‐rank (Mantel‐Cox) test. (b) Mean fluid intake (mL per day). *p*‐values were determined by One‐way ANOVA with Holm‐Šidák posttest. (c) Mean weight development. *p*‐values were determined by One‐way ANOVA with Holm‐Šidák posttest. (d) Ejection Fraction [%] 28 days after MI. *p*‐values were determined by Kruskal‐Wallis test with Dunn posttest. *n* = 4 per group**.** (e) Fractional Area Shortening [%] 28 days after MI. *p*‐values were determined by Kruskal‐Wallis test with Dunn posttest. *n* = 4 per group. (f) Left Ventricular Inner Diameter [μm] 28 days after MI. *p*‐values were determined by Kruskal‐Wallis test with Dunn posttest. *n* = 4 per group. (g) Representative echocardiographic images. All echocardiography parameters were quantified using 2D B‐Mode tracings with short‐ and long‐axis.

Since we previously found that mesalazine exerts antifibrotic effects in vitro and in vivo by modulating ERK1/2‐SMAD2/3‐, NFκB‐ and osteopontin‐signaling (Hoffmann et al., 2021; Künzel et al., 2021; Newe et al., 2021), we tested the effects of systemically administered mesalazine on cardiac function and fibrotic remodeling following MI. Kaplan–Meier analysis showed no significant difference in 28‐day‐survival of mesalazine‐treated mice compared to MI and Sham controls (Figure 2A). Fluid intake as a surrogate for drug intake via the drinking water as well as weight development were monitored closely throughout the experiment and showed no significant differences between the three groups (Figure 2B,C). Compared to Sham, echocardiography revealed impaired cardiac function in infarcted mice as determined by reduced ejection fraction (EF), reduced fractional area shortening (FAS), and dilation of the left ventricle (LVID) 14 and 28 days post MI (Figure 2D–G; a comprehensive list of all echocardiography results is provided in Table S1). Overall, mesalazine treatment did not significantly affect cardiac function compared to untreated mice with MI. Kidney function was assessed by plasma creatinine levels measured in blood samples taken at the end of the experiment. Mesalazine treatment did not negatively affect kidney function in treated mice (Figure S1).

### 3.2 Antifibrotic potential of mesalazine treatment in the infarcted heart

**FIGURE 3:**
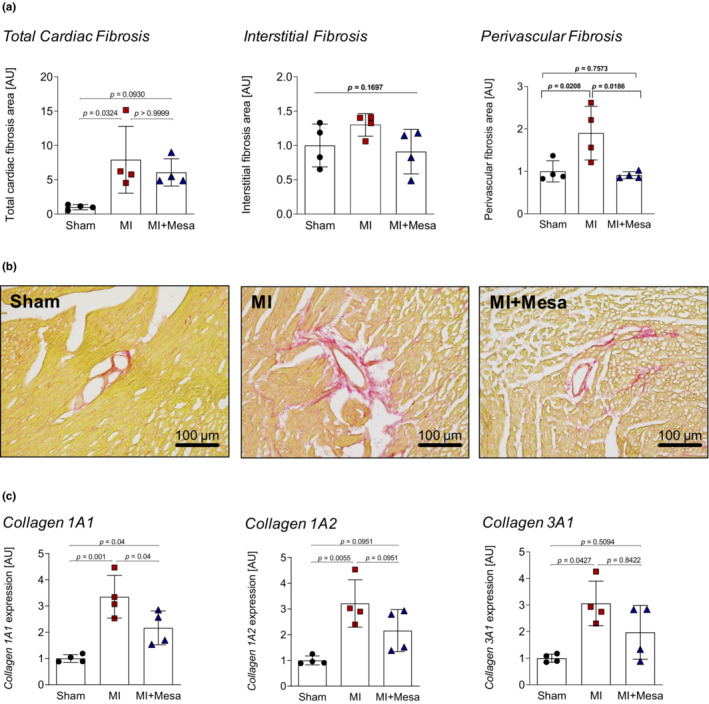
Mesalazine effects after myocardial infarction. The bar graphs represent the mean values of the indicated study populations normalized to the mean value of the respective control group ± SD. (a) Quantification of cardiac fibrosis after 28 days of mesalazine or control treatment. Left panel: total cardiac fibrosis. *p*‐values were determined by Kruskal‐Wallis test with Dunn posttest. *n* = 4 per group Mid panel: total cardiac fibrosis. *p*‐values were determined by One‐way ANOVA with Holm‐Šidák posttest. *n* = 4 per group. Randomly chosen interstitial areas of the histological section were analyzed and mean values were calculated for each animal. Right panel: Perivascular fibrosis. *p*‐values were determined by One‐way ANOVA with Holm‐Šidák posttest. *n* = 4 per group. Randomly chosen areas with blood vessels present were analyzed and mean values were calculated for each animal. (b) Representative Sirius Red collagen stainings of perivascular fibrosis areas. (c) Collagen gene expression analysis using qPCR. HPRT was used as the housekeeping gene. Collagen 1A1: *p*‐values were determined by Kruskal‐Wallis test with Dunn posttest. *n* = 4 per group. Collagen 1A2, 3A1: *p*‐values were determined by One‐way ANOVA with Holm‐Šidák posttest. *n* = 4 per group.

Next, we analyzed fibrosis development in mid‐ventricular whole‐heart sections (Figure 3A,B). As expected, MI led to significant fibrotic response (Figure 3A, left panel). The total fibrotic area, which consisted mainly of the infarct scar, as well as interstitial fibrosis, however, remained unaltered in the mesalazine treated group (Figure 3A, left and middle panel). Surprisingly, the analysis of the perivascular tissue revealed significantly lower levels of perivascular fibrosis, comparable to control levels (Figure 3A, right panel). Finally, we performed a gene expression analysis of the most relevant cardiac collagens in the context of fibrosis (Collier et al., 2012; Weber et al., 2013). Compared to MI, we found a significant reduction of *collagen 1A1* expression in the mesalazine‐treated animals. Additionally, there was no significant difference in *collagen 1A2* and *collagen 3A1* expression in the mesalazine‐treated animals compared to Sham. In contrast, the MI animals, which did not receive mesalazine treatment, displayed significantly higher *collagen 1A2* and *collagen 3A1* expression than Sham (Figure 3C).

## 4 DISCUSSION

MI induces a pronounced transient inflammatory reaction triggering fibroblast activation, which is required for effective scar formation and survival (van Amerongen et al., 2007). However, when (myo)fibroblast activity remains unremitted, progressive secretion of extracellular matrix proteins (ECM) and pro‐fibrotic mediators leads to spreading of diffuse fibrosis and impaired cardiac performance in the long run (Gibb et al., 2020). As post‐MI fibrotic remodeling is considered a leading cause for subsequent heart failure (Gibb et al., 2020), the discovery of novel antifibrotic pharmacotherapy approaches remains a yet unmet medical need. As our previous data on repurposed mesalazine indicated significant antifibrotic properties in vitro and in vivo (Hoffmann et al., 2021; Künzel et al., 2021; Newe et al., 2021), we aimed to elucidate, whether systemic mesalazine treatment is sufficient to reduce adverse fibrotic remodeling, and alleviate cardiac function and survival after MI.

Compared to solvent control, mesalazine treatment had neither adverse nor beneficial effects on cardiac function and survival after MI (Figure 2). Although a survival benefit would be desirable, it is important to note, that the critical wound healing phase after MI (Humeres & Frangogiannis, 2019; Talman & Ruskoaho, 2016), was not impaired either (Figure 2A). Nonetheless, based on the initial hypothesis that mesalazine might improve adverse structural remodeling following MI, additional beneficial effects of mesalazine treatment would expected to be in long‐term nature, that is, slowing the progression of heart failure, reducing symptoms, improving quality of life, and preventing arrhythmias (Heidenreich et al., 2022; Ponikowski et al., 2016; Roger, 2013), thus providing a secondary survival benefit. Those putative long‐term benefits remain to be investigated in future studies.

While total cardiac and interstitial fibrosis remained unaffected by mesalazine treatment, the development of perivascular fibrosis was prevented (Figure 3A). This finding was supported by a significant reduction in *collagen 1A1* expression, which is considered most relevant in cardiac fibrosis (Tallquist & Molkentin, 2017; Weber et al., 2013). Although the detrimental effects of cardiac fibrosis are known for many years, the impact of perivascular fibrosis on overall cardiac function, and patient outcome remained less clear. However, there is an emerging role for the perivascular niche in cardiac wound healing and scarring as perivascular mesenchymal cells have been suggested as a major driver of fibrosis in response to injury (Carlo & Peduto, 2018; Kramann et al., 2015). After MI, exaggerated collagen accumulation around small‐blood vessels contributes to tissue hypoxia (Ytrehus et al., 2018), and promotes further diffuse fibrotic remodeling (Künzel et al., 2021; Watson et al., 2014) leading to increased myocardial stiffness and diastolic dysfunction (Baum & Duffy, 2011; Burlew & Weber, 2002; Frangogiannis, 2021), thus limiting the long‐term prognosis of MI survivors (Frangogiannis, 2017; Talman & Ruskoaho, 2016). Although genetic interventions in cells surrounding the macro‐ and micro‐vasculature have shown promising antifibrotic effects in mice (Kramann et al., 2015), there are currently no clinically applicable pharmacological therapies directed at preventing cardiac perivascular fibrosis (Ytrehus et al., 2018).

The reason why mesalazine affected perivascular fibrosis in particular might be due to the pharmacological distribution of the hydrophilic compound. For the use of statins after MI, it has been demonstrated that the lipophilic atorvastatin offered advantages such as increasing left ventricular EF and reducing fibrosis marker expression over the hydrophilic rosuvastatin (El Said et al., 2021). The authors attribute this observation to increased extrahepatic tissue penetration of the lipophilic agent (El Said et al., 2021). Similar observations were made in the treatment of tuberculosis, in which lipophilic antibiotics displayed higher anti‐tuberculosis activity (Piccaro et al., 2015). Thus, we hypothesize that the bioavailability of hydrophilic mesalazine (Lau et al., 2013) might be greater around the blood vessels compared to deeper tissue levels and therefore the observed antifibrotic effects were concentrated in the proximity of the cardiac (micro)vasculature.

### 4.1 Potential limitations

Although the effects of mesalazine on perivascular fibrosis are intriguing, larger studies will be necessary to validate these findings. Measurements of crosslinked collagen and matrix metalloprotease expression could shed further light on the mechanisms of mesalazine action in the context of fibrosis and its effects on collagen accumulation. Additionally, a continuous drug administration for example, with osmotic micropumps (Herrlich et al., 2012) and several dosage steps would be preferable to ensure constant mesalazine levels. As myofibroblasts can persist in the infarcted heart for several years (Willems et al., 1994), and beneficial effects of anti‐remodeling drugs are expected to be in long‐term nature, studies looking at later time points after MI in aged animals will be necessary to determine definitive outcomes of mesalazine treatment following MI.

## 5 CONCLUSIONS

The present study is, to the best of our knowledge, the first to test the effects of systemically administered mesalazine after MI. Although mesalazine did not significantly improve survival and cardiac function, perivascular fibrosis, which represents a detrimental long‐term complication in cardiovascular patients (Dai et al., 2012), has been successfully prevented. Hence, our study confirms previously observed antifibrotic effects of mesalazine, and adds another facet to its antifibrotic spectrum of activity. Our results prompt further investigation of mesalazine and its derivatives in larger studies as a putative add‐on medication following MI to mitigate adverse fibrotic remodeling.

## AUTHOR CONTRIBUTIONS

Stephan R. Künzel conceived the study, planned and analyzed experiments, acquired funding and wrote the paper. Luise Winter, Maximilian Hoffmann, and Theresa A. Kant performed and analyzed experiments. Jessica Thiel and Romy Kronstein‐Wiedemann analyzed data. Erik Klapproth and Kristina Lorenz planned and performed experiments. Ali El‐Armouche planned and supervised experiments. Susanne Kämmerer planned and supervised experiments. All authors contributed to the initial manuscript preparation.

## FUNDING INFORMATION

The present study was funded by a “*MeDDrive Start*” grant provided by The Faculty of Medicine Carl Gustav Carus Dresden to SRK (no grant number available), by the German Research Foundation (DFG) grants EL 270/7–3, Transregio‐SFB CRC/TRR 205 to AEA, grant KA 4194/3–3 to SK, project no. 288034826—international research training group (IRTG) 2251 to AEA and SK.

## CONFLICT OF INTEREST STATEMENT

The authors declare that the research was conducted in the absence of any commercial or financial relationships that could be construed as a potential conflict of interest.

## ETHICS STATEMENT

Animal experiments were authorized by *Landesdirektion Sachsen*, Dresden, Germany, according to the German animal welfare regulations (TVV 64/2018) and complied with the ARRIVE guidelines as well as the guidelines from Directive 2010/63/EU on the protection of animals used for scientific purposes. For this study, male wild‐type C57BL/6J mice were obtained from Janvier Laboratories, Saint Berthevin Cedex, France.

## Supporting information


**Supplementary Table 1:** Investigation of mesalazine as an antifibrotic drug following myocardial infarction in male mice

**Supplementary Figure 1.** Plasma Creatinine Levels. Peripheral blood was collected at the end of the experiment. A colorimetric creatinine assay kit (ab65340, Abcam, Cambridge, UK) was used to determine respective plasma levels in the experimental groups. *n* = 4 samples from *N* = 4 animals per group.
Supplementary Table 1. Echocardiography data

## Data Availability

All data obtained in this study is depicted within the figures and tables.
