# Supplementary material for: Investigation of mesalazine as an antifibrotic drug following myocardial infarction in male mice
Source: Physiol Rep. 2023 Sep 9;11(17):e15809. doi: 10.14814/phy2.15809 (PMC10492006; doi:10.14814/phy2.15809)
Supplement: Supplementary file 2 — Supplementary Figure 1. Plasma Creatinine Levels. Peripheral blood was collected at the end of the experiment. A colorimetric creatinine assay kit (ab65340, Abcam, Cambridge, UK) was used to determine respective plasma levels in the experimental groups. n = 4 samples from N = 4 animals per group. Supplementary Table 1. Echocardiography data [file PHY2-11-e15809-s001.pdf]

Investigation of mesalazine as an antifibrotic drug following myocardial infarction in male mice

Stephan R. Künzel[1,2,3], Luise Winter[1], Maximilian Hoffmann[1], Theresa A. Kant[1], Jessica Thiel[2,3], Romy Kronstein-Wiedemann[2,3], Erik Klapproth[1], Kristina Lorenz[4,5], Ali El-Armouche[1], Susanne Kämmerer[1]

1 Institute of Pharmacology and Toxicology, Faculty of Medicine Carl Gustav Carus, Technische Universität Dresden, Dresden, Germany

2 Institute of Transfusion Medicine, Faculty of Medicine Carl Gustav Carus, Technische Universität Dresden, Dresden, Germany

3 German Red Cross Blood Donation Service North-East, Dresden, Germany

4 Institute of Pharmacology and Toxicology, Julius-Maximilians-University of Würzburg, Würzburg, Germany

5 Leibniz-Institut für Analytische Wissenschaften -ISAS- e.V., Dortmund, Germany

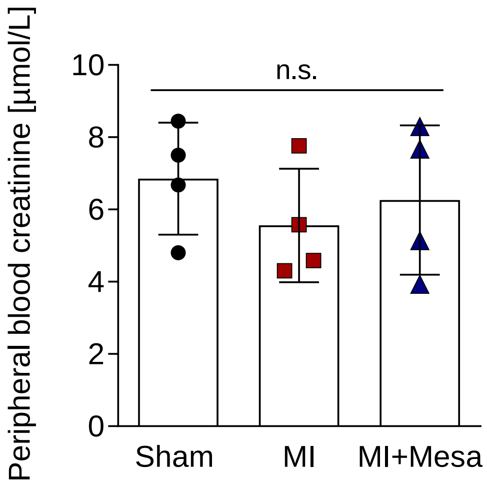

Supplementary Figure 1. Plasma Creatinine Levels. Peripheral blood was collected at the end of the experiment. A colorimetric creatinine assay kit (ab65340, Abcam, Cambridge, UK) was used to determine respective plasma levels in the experimental groups. n = 4 samples from N = 4 animals per group.

Supplementary Table 1. Echocardiography data

	SHAM Baseline									
**Animal No.**	**1**	**2**	**3**	**4**	**5**	**6**	**7**	**8**		
	♂	♂	♂	♂	♂	♂	♂	♂	**Mean**	**SD**
**B-Mode**										
**Ls [mm]**	7,21	7,19	7,59	7,34	7,31	7,10	7,11	7,32	7,27	0,16
**Ld [mm]**	8,26	8,21	8,39	7,93	7,82	8,18	7,79	8,33	8,11	0,23
**AWThd [mm]**	0,33	0,43	0,45	0,38	0,40	0,40	0,61	0,43	0,43	0,08
**LVIDd [mm]**	4,79	4,49	4,69	4,71	4,59	4,77	4,44	4,58	4,63	0,13
**PWThd [mm]**	0,23	0,33	0,23	0,28	0,23	0,33	0,25	0,21	0,26	0,05
**Area d [mm2]**	15,36	14,41	16,38	16,98	15,62	16,31	15,28	15,04	15,67	0,83
**AWThs [mm]**	0,61	0,61	0,76	0,83	0,71	0,88	0,79	0,85	0,75	0,11
**LVIDs [mm]**	3,71	3,46	3,51	3,68	3,45	3,33	3,50	3,23	3,48	0,16
**PWThs [mm]**	0,68	0,58	0,63	0,40	0,66	0,53	0,65	0,61	0,59	0,09
**Area s [mm2]**	8,75	8,52	8,88	9,77	8,59	8,44	9,86	7,78	8,82	0,69
**Epi s [mm]**	17,23	17,58	18,76	18,29	17,34	17,82	19,62	17,86	18,06	0,80
**HR [bpm]**	388,00	426,00	418,00	453,00	436,00	480,00	467,00	466,00	441,75	30,59
**RR [1/min]**	85,00	87,00	82,00	108,00	67,00	124,00	63,00	60,00	84,50	22,34
**BT [°C]**	36,20	36,40	36,50	36,80	35,80	36,00	36,30	36,20	36,28	0,31
**BW [g]**	23,50	22,10	24,40	23,50	23,00	25,00	23,10	24,80	23,68	0,99
**B-Mode**										
**FAS [%]**	43,06	40,85	45,80	42,47	45,02	48,26	35,48	48,30	43,66	4,24
**AWThF [%]**	45,71	29,21	39,95	54,62	42,92	54,31	22,93	50,00	42,46	11,46
**PWThF [%]**	66,67	43,28	64,08	31,44	65,40	37,81	62,15	64,79	54,45	14,44
**Vol s [µl]**	52,52	51,07	56,12	59,71	52,32	49,93	58,39	47,44	53,44	4,26
**Vol d [µl]**	105,77	98,57	114,43	112,18	101,74	111,22	99,14	104,40	105,93	6,09
**EF [%]**	50,35	48,19	50,96	46,77	48,58	55,11	41,10	54,56	49,45	4,48
**SV [µl]**	53,26	47,51	58,31	52,47	49,42	61,29	40,75	56,96	52,50	6,59
**CO [ml/min]**	20,66	20,24	24,38	23,77	21,55	29,42	19,03	26,54	23,20	3,52
**CI [ml/min/g]**	879,29	915,74	998,99	1011,38	936,89	1176,77	823,86	1070,27	976,65	112,52

	MI BASELINE													
**Animal No.**	**10**	**12**	**17**	**20**	**9**	**11**	**13**	**14**	**15**	**16**	**18**	**19**		
	♂	♂	♂	♂	♂	♂	♂	♂	♂	♂	♂	♂	**Mean**	**SD**
**B-Mode**														
**Ls [mm]**	7,31	7,10	7,53	7,02	7,10	7,43	7,27	6,75	7,27	7,02	7,34	7,24	7,20	0,21
**Ld [mm]**	7,80	7,78	8,44	7,56	7,89	8,11	8,09	7,66	8,05	7,67	7,99	8,27	7,94	0,26
**AWThd [mm]**	0,66	0,28	0,34	0,25	0,45	0,38	0,40	0,38	0,45	0,45	0,35	0,25	0,39	0,11
**LVIDd [mm]**	4,39	4,84	4,69	4,87	4,64	4,22	4,73	4,28	4,71	4,58	4,74	4,77	4,62	0,21
**PWThd [mm]**	0,38	0,18	0,23	0,20	0,30	0,29	0,25	0,22	0,17	0,18	0,28	0,20	0,24	0,06
**Area d [mm2]**	14,91	16,71	15,07	16,50	15,99	13,14	15,08	13,83	16,14	15,02	15,87	15,68	15,33	1,06
**AWThs [mm]**	1,01	0,56	0,83	0,58	0,83	0,72	0,76	0,72	0,67	0,71	0,76	0,71	0,74	0,12
**LVIDs [mm]**	3,08	3,74	3,13	3,76	3,53	3,03	3,27	3,03	3,95	3,61	3,81	3,56	3,46	0,34
**PWThs [mm]**	0,94	0,58	0,85	0,53	0,78	0,76	0,61	0,87	0,51	0,43	0,51	0,56	0,66	0,17
**Area s [mm2]**	6,70	10,27	6,84	10,42	9,01	6,50	8,04	6,68	10,85	9,34	9,40	8,73	8,56	1,59
**Epi s [mm]**	18,08	18,78	17,54	18,05	20,68	16,27	16,81	16,14	19,07	17,12	18,82	17,20	17,88	1,31
**HR [bpm]**	434,00	424,00	447,00	442,00	358,00	453,00	354,00	437,00	421,00	448,00	442,00	461,00	426,75	34,91
**RR [1/min]**	60,00	106,00	84,00	110,00	67,00	66,00	69,00	96,00	87,00	89,00	91,00	82,00	83,92	15,97
**BT [°C]**	36,60	36,40	36,10	36,90	35,20	36,10	35,60	36,90	36,60	36,70	36,90	36,50	36,38	0,54
**BW [g]**	24,40	24,30	25,50	23,20	25,20	24,00	21,60	22,60	21,70	23,80	24,30	24,00	23,72	1,23
**B-Mode**														
**FAS [%]**	55,05	38,52	54,63	36,88	43,69	50,53	46,70	51,69	32,75	37,77	40,76	44,34	44,44	7,38
**AWThF [%]**	35,11	50,09	59,40	56,55	45,43	46,86	46,98	46,86	33,18	35,69	53,37	64,41	47,83	9,78
**PWThF [%]**	59,51	69,42	73,59	61,63	61,25	61,68	59,01	74,37	66,47	58,51	45,15	63,42	62,83	7,79
**Vol s [µl]**	40,80	60,79	42,89	60,90	53,27	40,26	48,67	37,59	65,76	54,69	57,48	52,63	51,31	9,26
**Vol d [µl]**	96,85	108,28	105,92	103,95	105,11	88,74	101,64	88,19	108,23	96,02	105,60	108,09	101,38	7,27
**EF [%]**	57,87	43,86	59,51	41,41	49,32	54,63	52,11	57,38	39,24	43,05	45,57	51,31	49,60	6,93
**SV [µl]**	56,05	47,49	63,03	43,05	51,84	48,48	52,97	50,60	42,47	41,34	48,12	55,46	50,07	6,33
**CO [ml/min]**	24,32	20,14	28,17	19,03	18,56	21,96	18,75	22,11	17,88	18,52	21,27	25,57	21,36	3,24
**CI [ml/min/g]**	996,92	828,62	1104,88	820,14	736,49	915,04	868,07	978,42	823,93	778,07	875,28	1065,30	899,26	115,03

	MI + Mesa BASELINE													
**Animal No.**	**27**	**28**	**29**	**31**	**21**	**22**	**23**	**24**	**25**	**26**	**30**	**32**		
	♂	♂	♂	♂	♂	♂	♂	♂	♂	♂	♂	♂	**Mean**	**SD**
**B-Mode**														
**Ls [mm]**	7,32	7,28	7,26	7,08	7,10	7,32	7,60	6,99	6,95	7,13	7,02	7,80	7,24	0,25
**Ld [mm]**	7,83	7,96	8,60	7,83	7,98	7,97	8,35	7,68	7,98	7,62	7,82	8,69	8,02	0,34
**AWThd [mm]**	0,38	0,38	0,34	0,38	0,34	0,31	0,45	0,43	0,36	0,38	0,30	0,50	0,38	0,06
**LVIDd [mm]**	4,71	4,53	4,91	4,51	4,33	4,30	4,46	4,36	4,53	4,53	4,59	4,17	4,49	0,20
**PWThd [mm]**	0,23	0,25	0,20	0,23	0,18	0,36	0,34	0,30	0,27	0,25	0,30	0,34	0,27	0,06
**Area d [mm2]**	15,40	14,81	16,92	14,44	13,06	13,06	14,81	13,62	13,89	14,08	14,59	12,54	14,27	1,19
**AWThs [mm]**	0,61	0,74	0,58	0,71	0,63	0,70	0,91	0,73	0,58	0,70	0,66	0,78	0,69	0,09
**LVIDs [mm]**	3,71	3,57	4,01	3,38	3,41	3,15	3,17	3,33	3,29	3,72	3,61	2,76	3,43	0,33
**PWThs [mm]**	0,58	0,61	0,45	0,63	0,56	0,81	0,84	0,66	0,70	0,65	0,71	0,83	0,67	0,12
**Area s [mm2]**	10,30	9,35	10,89	8,09	7,95	6,97	6,89	7,40	7,64	9,06	8,84	5,31	8,22	1,56
**Epi s [mm]**	18,11	17,63	18,24	17,48	16,16	15,79	17,26	16,52	16,44	17,24	17,68	15,23	16,98	0,94
**HR [bpm]**	445,00	452,00	430,00	479,00	418,00	472,00	472,00	479,00	390,00	451,00	429,00	460,00	448,08	27,30
**RR [1/min]**	72,00	106,00	100,00	103,00	88,00	95,00	85,00	89,00	94,00	109,00	137,00	75,00	96,08	17,24
**BT [°C]**	36,70	37,00	36,40	36,40	36,10	36,80	37,10	36,80	35,80	37,10	36,70	36,00	36,58	0,44
**BW [g]**	24,70	22,50	24,70	22,00	22,20	24,10	23,10	21,90	23,30	22,20	22,80	25,60	23,26	1,24
**B-Mode**														
**FAS [%]**	33,12	36,84	35,64	43,98	39,13	46,64	53,45	45,66	45,00	35,66	39,39	57,66	42,68	7,51
**AWThF [%]**	37,52	48,44	42,20	46,46	46,50	54,82	50,77	41,31	38,42	45,04	53,74	36,86	45,17	6,10
**PWThF [%]**	60,86	59,34	54,69	63,87	67,91	55,57	59,86	53,81	61,15	62,15	57,06	59,47	59,64	4,02
**Vol s [µl]**	62,81	56,70	65,93	47,75	47,02	42,52	43,66	43,08	44,26	53,81	51,76	34,51	49,48	9,09
**Vol d [µl]**	100,47	98,20	121,28	94,20	86,81	86,66	102,97	87,20	92,31	89,40	95,12	90,80	95,45	9,75
**EF [%]**	37,48	42,26	45,64	49,31	45,83	50,94	57,60	50,59	52,06	39,81	45,58	61,99	48,26	7,05
**SV [µl]**	37,65	41,50	55,35	46,45	39,78	44,15	59,31	44,12	48,05	35,59	43,36	56,29	45,97	7,54
**CO [ml/min]**	16,76	18,76	23,80	22,25	16,63	20,84	27,99	21,13	18,74	16,05	18,60	25,89	20,62	3,79
**CI [ml/min/g]**	678,38	833,78	963,66	1011,34	749,09	864,63	1211,85	964,90	804,33	722,94	815,84	1011,40	886,01	151,68

	SHAM 14d post MI						
**Animal No.**	**1**	**2**	**3**	**4**	**5**		
	♂	♂	♂	♂	♂	**Mean**	**SD**
**B-Mode**							
**Ls [mm]**	7,55	7,74	7,26	7,52	7,12	7,44	0,25
**Ld [mm]**	8,38	8,45	8,21	8,04	7,87	8,19	0,24
**AWThd [mm]**	0,40	0,36	0,63	0,38	0,52	0,46	0,11
**LVIDd [mm]**	4,89	4,26	4,44	4,74	4,31	4,53	0,28
**PWThd [mm]**	0,23	0,27	0,40	0,30	0,34	0,31	0,07
**Area d [mm2]**	17,09	14,07	15,27	16,52	14,09	15,41	1,38
**AWThs [mm]**	0,73	0,52	0,91	0,83	0,94	0,79	0,17
**LVIDs [mm]**	3,73	3,68	3,30	3,66	3,14	3,50	0,26
**PWThs [mm]**	0,53	0,65	0,68	0,63	0,61	0,62	0,06
**Area s [mm2]**	10,41	9,26	7,75	9,11	7,09	8,72	1,31
**Epi s [mm]**	19,58	16,26	18,93	19,75	16,95	18,29	1,59
**HR [bpm]**	403,00	433,00	411,00	400,00	386,00	406,60	17,30
**RR [1/min]**	70,00	56,00	72,00	80,00	71,00	69,80	8,67
**BT [°C]**	36,50	35,10	35,60	35,70	35,80	35,74	0,50
**BW [g]**	24,90	24,20	25,50	26,20	24,30	25,02	0,84
**B-Mode**							
**FAS [%]**	39,10	34,18	49,25	44,89	49,66	43,41	6,69
**AWThF [%]**	44,81	30,43	30,51	54,57	45,27	41,12	10,47
**PWThF [%]**	57,09	58,62	40,82	51,75	44,65	50,58	7,73
**Vol s [µl]**	65,52	59,74	46,93	57,08	42,06	54,26	9,58
**Vol d [µl]**	119,39	99,09	104,54	110,70	92,43	105,23	10,40
**EF [%]**	45,12	39,72	55,11	48,44	54,50	48,58	6,48
**SV [µl]**	53,87	39,36	57,61	53,62	50,37	50,97	6,98
**CO [ml/min]**	21,71	17,04	23,68	21,45	19,44	20,66	2,52
**CI [ml/min/g]**	871,83	704,21	928,58	818,68	800,16	824,69	83,92

	MI 14d post MI					
**Animal No.**	**10**	**12**	**17**	**20**		
	♂	♂	♂	♂	**Mean**	**SD**
**B-Mode**						
**Ls [mm]**	7,62	7,99	8,95	7,28	7,96	0,72
**Ld [mm]**	8,11	8,30	9,15	7,70	8,32	0,61
**AWThd [mm]**	0,06	0,09	0,09	0,05	0,07	0,02
**LVIDd [mm]**	5,77	6,00	6,88	6,05	6,17	0,49
**PWThd [mm]**	0,50	0,39	0,38	0,37	0,41	0,06
**Area d [mm2]**	26,77	24,85	31,30	26,18	27,28	2,80
**AWThs [mm]**	0,09	0,11	0,12	0,10	0,11	0,01
**LVIDs [mm]**	5,21	5,75	6,40	5,72	5,77	0,49
**PWThs [mm]**	0,79	0,59	0,59	0,58	0,64	0,10
**Area s [mm2]**	20,48	21,29	27,03	22,86	22,92	2,92
**Epi s [mm]**	28,79	27,99	34,28	30,37	30,36	2,79
**HR [bpm]**	380,00	466,00	551,00	350,00	436,75	90,65
**RR [1/min]**	50,00	66,00	60,00	90,00	66,50	17,00
**BT [°C]**	35,40	36,10	35,60	34,60	35,43	0,62
**BW [g]**	26,70	25,60	26,80	24,60	25,93	1,04
**B-Mode**						
**FAS [%]**	23,50	14,32	13,63	12,66	16,03	5,03
**AWThF [%]**	29,21	20,54	25,20	53,85	32,20	14,86
**PWThF [%]**	35,88	33,16	35,75	34,96	34,94	1,25
**Vol s [µl]**	130,05	141,73	201,65	138,61	153,01	32,80
**Vol d [µl]**	180,97	171,96	238,75	168,00	189,92	33,00
**EF [%]**	28,13	17,58	15,54	17,49	19,68	5,71
**SV [µl]**	50,91	30,22	37,10	29,38	36,90	9,96
**CO [ml/min]**	19,35	14,08	20,44	10,28	16,04	4,73
**CI [ml/min/g]**	724,60	550,12	762,71	418,07	613,88	160,02

	MI + Mesa 14d post MI					
**Animal No.**	**27**	**28**	**29**	**31**		
	♂	♂	♂	♂	**Mean**	**SD**
**B-Mode**						
**Ls [mm]**	7,57	7,66	8,86	8,06	8,04	0,59
**Ld [mm]**	7,69	7,93	8,92	8,18	8,18	0,53
**AWThd [mm]**	0,12	0,06	0,09	0,07	0,09	0,02
**LVIDd [mm]**	6,67	5,63	6,58	6,18	6,26	0,47
**PWThd [mm]**	0,28	0,35	0,22	0,40	0,31	0,08
**Area d [mm2]**	35,57	21,83	30,88	29,62	29,48	5,70
**AWThs [mm]**	0,138	0,100	0,164	0,100	0,13	0,03
**LVIDs [mm]**	6,61	5,02	6,33	5,77	5,93	0,70
**PWThs [mm]**	0,42	0,47	0,32	0,52	0,43	0,09
**Area s [mm2]**	32,15	16,71	26,63	25,30	25,20	6,39
**Epi s [mm]**	39,40	23,57	34,54	32,87	32,60	6,62
**HR [bpm]**	508,00	364,00	508,00	520,00	475,00	74,22
**RR [1/min]**	65,00	98,00	77,00	89,00	82,25	14,36
**BT [°C]**	35,30	35,20	35,20	35,70	35,35	0,24
**BW [g]**	26,00	24,30	24,30	25,00	24,90	0,80
**B-Mode**						
**FAS [%]**	9,60	23,47	13,78	14,58	15,36	5,83
**AWThF [%]**	13,77	38,00	42,68	29,00	30,86	11,03
**PWThF [%]**	32,86	26,28	29,87	24,24	28,31	3,82
**Vol s [µl]**	202,89	106,62	196,66	169,98	169,04	43,99
**Vol d [µl]**	228,02	144,21	229,51	201,90	200,91	39,87
**EF [%]**	11,02	26,07	14,31	15,81	16,80	6,49
**SV [µl]**	25,14	37,60	32,85	31,92	31,87	5,14
**CO [ml/min]**	12,77	13,69	16,69	16,60	14,93	2,01
**CI [ml/min/g]**	491,10	563,17	686,73	663,84	601,21	90,94

	SHAM 28d post MI					
**Animal No.**	**1**	**3**	**4**	**5**		
	♂	♂	♂	♂	**Mean**	**SD**
**B-Mode**						
**Ls [mm]**	6,91	7,67	7,31	7,36	7,31	0,31
**Ld [mm]**	8,18	8,38	8,36	8,25	8,29	0,10
**AWThd [mm]**	0,31	0,56	0,58	0,59	0,51	0,13
**LVIDd [mm]**	4,84	4,77	4,44	4,47	4,63	0,20
**PWThd [mm]**	0,27	0,35	0,35	0,42	0,35	0,06
**Area d [mm2]**	16,85	16,75	15,18	16,81	16,39	0,81
**AWThs [mm]**	0,76	0,98	0,93	0,77	0,86	0,11
**LVIDs [mm]**	3,63	3,53	3,41	3,82	3,60	0,17
**PWThs [mm]**	0,67	0,91	0,86	0,54	0,74	0,17
**Area s [mm2]**	9,04	8,48	8,81	9,65	8,99	0,49
**Epi s [mm]**	19,09	21,51	22,06	20,15	20,70	1,34
**HR [bpm]**	360,00	468,00	470,00	358,00	414,00	63,52
**RR [1/min]**	68,00	66,00	112,00	54,00	75,00	25,43
**BT [°C]**	35,10	36,20	36,60	35,20	35,78	0,74
**BW [g]**	25,40	26,90	26,60	26,10	26,25	0,66
**B-Mode**						
**FAS [%]**	46,32	49,36	41,96	42,61	45,06	3,45
**AWThF [%]**	58,79	43,60	37,83	23,14	40,84	14,75
**PWThF [%]**	60,09	61,01	58,69	22,08	50,47	18,95
**Vol s [µl]**	52,07	54,20	53,64	59,19	54,78	3,08
**Vol d [µl]**	114,83	117,01	105,72	115,49	113,26	5,11
**EF [%]**	54,65	53,68	49,26	48,75	51,58	3,01
**SV [µl]**	62,75	62,81	52,08	56,30	58,48	5,25
**CO [ml/min]**	22,59	29,39	24,48	20,15	24,15	3,92
**CI [ml/min/g]**	889,40	1092,69	920,23	772,20	918,63	132,41
**HW [g]**	0,141	0,163	0,135	0,128	0,142	0,013
**HW/BW**	0,006	0,006	0,005	0,005	0,005	0,000
**HW/Tibia Length [g/cm]**	0,109	0,130	0,117	0,093	0,112	0,014

	MI 28d post MI					
**Animal No.**	**10**	**12**	**17**	**20**		
	♂	♂	♂	♂	**Mean**	**SD**
**B-Mode**						
**Ls [mm]**	8,07	7,78	8,57	7,16	7,89	0,59
**Ld [mm]**	8,54	7,81	9,31	7,72	8,34	0,74
**AWThd [mm]**	0,18	0,06	0,06	0,12	0,11	0,05
**LVIDd [mm]**	5,92	6,21	6,76	6,02	6,22	0,37
**PWThd [mm]**	0,37	0,35	0,32	0,33	0,34	0,02
**Area d [mm2]**	26,78	27,68	29,92	25,19	27,39	1,97
**AWThs [mm]**	0,20	0,09	0,08	0,19	0,14	0,06
**LVIDs [mm]**	5,67	5,62	6,24	5,56	5,77	0,31
**PWThs [mm]**	0,44	0,60	0,76	0,54	0,58	0,14
**Area s [mm2]**	23,08	21,99	25,48	21,93	23,12	1,66
**Epi s [mm]**	29,53	29,05	35,14	29,44	30,79	2,91
**HR [bpm]**	441,00	437,00	423,00	412,00	428,25	13,30
**RR [1/min]**	98,00	68,00	95,00	85,00	86,50	13,53
**BT [°C]**	36,10	35,70	35,30	36,00	35,78	0,36
**BW [g]**	28,30	26,90	27,80	25,30	27,08	1,32
**B-Mode**						
**FAS [%]**	13,83	20,56	14,83	12,93	15,53	3,44
**AWThF [%]**	11,06	28,09	23,46	36,08	24,67	10,47
**PWThF [%]**	14,02	42,10	57,76	38,78	38,16	18,10
**Vol s [µl]**	155,22	142,54	181,88	130,80	152,61	21,92
**Vol d [µl]**	190,59	180,16	232,02	162,01	191,20	29,67
**EF [%]**	18,56	20,88	21,61	19,27	20,08	1,41
**SV [µl]**	35,37	37,62	50,14	31,21	38,59	8,14
**CO [ml/min]**	15,60	16,44	21,21	12,86	16,53	3,47
**CI [ml/min/g]**	551,24	611,21	762,87	508,31	608,41	111,29
**HW [g]**	0,213	0,233	0,201	0,195	0,210	0,015
**HW/BW**	0,008	0,009	0,007	0,008	0,008	0,001
**HW/Tibia Length [g/cm]**	0,152	0,173	0,157	0,150	0,158	0,009

	MI + Mesa 28d post MI					
**Animal No.**	**27**	**28**	**29**	**31**		
	♂	♂	♂	♂	**Mean**	**SD**
**B-Mode**						
**Ls [mm]**	8,13	7,66	9,23	8,03	8,26	0,68
**Ld [mm]**	8,26	7,93	9,30	8,13	8,40	0,61
**AWThd [mm]**	0,15	0,04	0,10	0,10	0,10	0,04
**LVIDd [mm]**	6,86	5,98	7,09	6,20	6,53	0,53
**PWThd [mm]**	0,23	0,35	0,34	0,53	0,36	0,12
**Area d [mm2]**	37,55	24,25	34,54	31,81	32,04	5,70
**AWThs [mm]**	0,20	0,06	0,13	0,13	0,13	0,05
**LVIDs [mm]**	6,75	5,29	6,85	6,12	6,25	0,72
**PWThs [mm]**	0,26	0,47	0,43	0,57	0,43	0,13
**Area s [mm2]**	34,09	17,65	33,58	26,72	28,01	7,68
**Epi s [mm]**	40,58	23,83	40,12	32,33	34,22	7,89
**HR [bpm]**	511,00	392,00	504,00	529,00	484,00	62,23
**RR [1/min]**	82,00	88,00	88,00	82,00	85,00	3,46
**BT [°C]**	36,60	36,20	35,40	36,10	36,08	0,50
**BW [g]**	27,80	25,90	27,30	26,80	26,95	0,81
**B-Mode**						
**FAS [%]**	9,22	27,20	2,79	16,00	13,81	10,43
**AWThF [%]**	22,05	32,81	19,69	21,09	23,91	6,01
**PWThF [%]**	9,80	26,37	21,53	8,22	16,48	8,87
**Vol s [µl]**	230,89	112,62	258,23	178,72	195,12	64,13
**Vol d [µl]**	258,36	160,14	267,74	215,53	225,44	49,11
**EF [%]**	10,63	29,67	3,55	17,08	15,23	11,10
**SV [µl]**	27,47	47,52	9,51	36,81	30,33	16,11
**CO [ml/min]**	14,04	18,63	4,80	19,47	14,23	6,73
**CI [ml/min/g]**	504,90	719,17	175,65	726,61	531,58	258,60
**HW [g]**	0,293	0,162	0,282	0,228	0,241	0,052
**HW/BW**	0,011	0,006	0,010	0,009	0,009	0,002
**HW/Tibia Length [g/cm]**	0,266	0,141	0,226	0,190	0,206	0,046
